# Determining the Optimal Cognitive Screening Tool in Older People With Diabetes

**DOI:** 10.3389/fendo.2020.00322

**Published:** 2020-05-22

**Authors:** Nadia Mordenfeld, Noa Gayus, Michal Azmon, Omri Guri-Twito, Tal Yahalom Peri, Rachel Natovich, Tali Cukierman-Yaffe

**Affiliations:** ^1^The Center for Successful Aging With Diabetes, Sheba Medical Center, Endocrinology Institute, Rama Gan, Israel; ^2^The Epidemiology Department, Sackler School of Medicine, Tel-Aviv University, Tel Aviv-Yafo, Israel; ^3^The Physiotherapy Department, Faculty of Health Sciences, Ariel University, Ariel, Israel; ^4^The Rehabilitation Hospital, Sheba Medical Center, Rama Gan, Israel; ^5^The Epidemiology Department, Sackler School of Medicine, Herczeg Institute on Aging, Tel-Aviv University, Tel Aviv-Yafo, Israel

**Keywords:** diabetes, elderly, cognition, cognitive assessment, self-care

## Abstract

**Background:** Self-care is an important perspective to aging and transitional states in diabetes management. Population studies have shown that lower cognitive function is associated with worse self-care abilities. Several guidelines have emphasized the importance of assessing cognitive function in older people with diabetes and tailoring treatment plan accordingly. Those guidelines do not specify which tools are the most appropriate for this population. One approach to delineate which tools should be used is to assess which tools best correlate with self-care capacity.

**Objective:** To assess which cognitive assessment tools best correlate with self-care capacity in older people with type 2 diabetes.

**Methods:** Cross-sectional study, conducted amongst individuals with diabetes over the age of 60. The association between self-care capacity indices and different cognitive assessment tools was examined. Principal Component self-care constructs were determined and the association between these and the different cognitive assessment tools was examined.

**Results:** A significant association was found between the Principal Component self-care construct and the Montreal Cognitive Assessment and Mindstreams^TM^ scores. In a stepwise regression model including only the Montreal Cognitive Assessment score, a significant association was found between this score and the Principal Component self-care construct. The same was not found in a model that included only the Mindstreams^TM^ scores.

**Conclusions:** The Montreal Cognitive Assessment, previously validated as a brief cognitive screening tool, may be useful as an adjunct to assess the self-care capacity of older individuals with diabetes. Future studies in the clinic are needed to evaluate if using this tool may improve treatment plans.

## Introduction

Self-care is a cornerstone in diabetes management and may be one of the important abilities lost as an individual moves between transition states. Involving a variety of health-promoting behaviors, such as appropriate diet and physical activity, self-care management has been shown to have positive effects on glucose control and in the prevention of the long-term consequences of diabetes (1–3). Cognitive dysfunction can potentially present new barriers to self-care and to achieving glycemic control. Indeed, population studies have shown that among people with diabetes, lower cognitive function was associated with worse efficacy of treatment indices, such as glucose control (4) and a greater risk for incident hypoglycemia (5). However, people with diabetes have an almost two-fold greater risk for developing cognitive impairment and dementia, compared to people without diabetes (6, 7).

The American Diabetes Association (ADA), as well as a number of published guidelines (8–12), have recently emphasized the importance of assessing cognitive function in older people with diabetes and tailoring treatment plan accordingly. However, those guidelines do not specify which tools are the most appropriate for cognitive evaluation in this population. One approach to delineate which tools should be used is to assess which tools best correlate with self-care capacity. The preferred tool should be freely available and easily administered, with minimal training and with validated translations to many languages.

Self-care was defined by the World Health Organization (WHO) as monitoring and responding to environmental and biological conditions by making adaptive adjustments in the different aspects of diabetes treatment (13). Various methods have been reported in order to assess self-care capacity (14). These methods rely either on self-reported questionnaires (that describe patients' adherence to different diabetes care domains) or on clinical outcomes (for example: glycated hemoglobin).

At the Center for Successful Aging with Diabetes, a multi-disciplinary evaluation is conducted. This includes collection of cognitive assessment and self-care capacity indices (based on self-reported questionnaires as well as on clinical outcomes) among people with diabetes over the age of 60. This evaluation provides us an opportunity to assess the association between self-care capacity indices and different cognitive instruments commonly used.

## Research Design and Methods

### General

This was a cross-sectional study conducted in individuals with diabetes over the age of 60, attending the Center for Successful Aging with Diabetes at the Sheba Medical Center (15). The individuals participated in an assessment day during which each individual was examined by a multi-disciplinary team of specialists, including neuropsychologist, physiotherapist, physician and dietitian. The study was approved by the ethical committee of the Sheba Medical Center and all participants signed a consent form.

### Population and Procedure

Hebrew speaking individuals with diagnosis of type 2 diabetes over the age of 60, either self-referred or referred by treating physician because of difficulties in managing their disease, were included. Diabetes diagnosis was determined by a diabetes specialist, based on medical history and diabetes medication. Excluded were people with significant hearing, visual, motor or cognitive impairment that may had precluded neuropsychological testing and responding to self-report questionnaires. Others exclusion criteria included illiteracy, any major non-diabetes related illness expected to reduce life expectancy or a significant disability that interfere with study participation.

### Measurement Tools and Instruments

- **Self-Care Capacity**
  The following variables were considered to be part of the self-care capacity construct:
  **(a)** *Summary of Diabetes Self-Care Activities Assessment* (SDSCA) (16) questionnaire components (including diabetes-specific diet, physical activity, blood-glucose testing, medication and foot care). The SDSCA validation is based on data from seven different studies, involving a total of 1,988 typically older people with diabetes; (**b)** The score on a *physical activity questionnaire* (PAQ), based on the *Baecke* (17) questionnaire; (**c)** Carbohydrate consumption/total caloric intake, according to a *Food Frequency Questionnaire* (FFQ) validated for the older population (18); (**d)** Report of whether or not the individual ever experienced an episode of severe hypoglycemia, requiring external assistance for recovery, as defined by the ADA (5); (**e)** Hemoglobin A1c (HbA1c).
- **Cognitive Function**
  Cognitive assessment was performed for each participant individually by a neuropsychologist. Cognitive function was measured using the *Mindstreams*^TM^ (NeuroTrax) (19) computerized neuropsychological battery tests, as well as paper-and-pencil tests. NeuroTrax is a computerized battery of tests, contains a set of tests designed for early detection of mild cognitive impairment (MCI) and mild dementia. Results from those tests were processed to form a global cognitive score (GCS), which is the mean of the cognitive domains examined, as well as scores in four specific cognitive domains: memory (mean accuracies for learning and delayed recognition phases of verbal and non-verbal memory tests), executive function (the ability to postpone an automatic response and to create a strategy to cope with a new task), attention (assessed using different tasks, including a timed continuous performance test during which responses are made to large colored stimuli that are any color but red) and motor skills (the ability to generate a motor response in an efficient manner). All scores were normalized to a standard distribution ($\bar{x}$ 100; σ 15), according to the expected performance by age and education years.
  Additionally, the following paper-and-pencil tests were also included: (a) *The Montreal Cognitive Assessment* (MoCA) (20), a brief cognitive screening instrument, assessing several cognitive domains, including attention, executive functions, language, memory, and orientation, with a maximum score of 30 points. The MoCA is composed of several subtasks: Delayed recall of 5 nouns (short-term memory); Clock-drawing task (visuospatial ability); Alternation task adapted from the Trail Making B task, phonemic fluency task and a two-item verbal abstraction task (executive functions); Sustained attention task, serial subtraction task and digits forward and backward (attention, concentration and working memory); Three-item confrontation naming task and repetition of two syntactically complex sentenced (language); (b) *The Digit Symbol Substitution Test* (DSST) (21), a subset of the Wechsler Adult Intelligence Scale (WAIS-III) (22), pertaining to a wide array of cognitive domains. This test has been extensively used among cognitively intact individuals, and its score is well correlated with measures of physical function and future cognitive decline (23, 24). Age standardized scores were used; (c) *Verbal Fluency Test* (VF), that measures verbal production, semantic memory and language (25). This test has been used in several longitudinal studies, exhibiting an ability to differentiate between people with and without diabetes, with respect to the rate of cognitive decline experienced over time (26, 27). Age standardized Semantic score and Phonetic score were processed from the results of this test.
- **Other Covariates**
  General, medical and diabetes related data variables were collected by a diabetes specialist, through history, physical examination, and blood work.

### Definitions and Statistical Analysis

All data was coded and unified into a common database. Continuous variables were summarized using means and standard deviations (SD)/medians and interquartile ranges, binary variables were summarized using counts and percentages. SDSCA diet and exercise domains were analyzed as continuous variables. Other SDSCA domains were dichotomized into “daily”/“less than daily” (blood-glucose testing and medication domains) or by their median (foot-care domain). To evaluate the association between each self-care capacity component and the cognitive evaluation tools, linear regressions were conducted on normally distributed self-care variables and logistic regression on other variables.

To examine the configuration of self-care capacity data in a multivariable space, Principal Component Analysis (PCA) was conducted. This statistical method was used to reduce the set of inter-correlated self-care capacity variables into a few dimensions, that gather as big amount as possible of the original variables variability (28). Using two sets of self-care capacity variables, different combinations of variables were analyzed into Principal Components self-care constructs (PCs), each representing a group of variables from the original data set. PCs are mathematical constructs, extracted as a linear combination of the self-care capacity variables and estimated from the correlation matrix. For mathematical reasons, using the correlation matrix in this procedure is equivalent to standardizing the variables to zero mean and unit standard deviation (29). PCs loadings, also known as eigenvectors, measure the correlation between the original self-care capacity variables and the constructed PCs. These loadings represent the importance of each variable in accounting for the total variability of self-care capacity represented in each PC. PCs loadings ≥0.35 are considered as significant contributors, while loadings ≥0.5 are referred as the main contributors to each PC (30). Loadings ≥ 0.6 are referred as highly associated contributors. Only PCs explaining more variance than a single variable are considered to simplify the data [“eigenvalues >one” criteria (31)], thus were used for further analysis.

We used stepwise linear regression to assess which cognitive assessment tools were significantly associated with each PC self-care construct, adjusted for age and gender. Further analysis was performed in separate regression models, including only cognitive assessment tools that were found to be significantly associated with the PC self-care construct. Statistical analysis was performed using version 3.5.0 of R statistical software (17).

## Results

### Participant Characteristics

The analysis pertains to 122 consecutive participants who conducted the evaluation day at the Center for Successful Aging with Diabetes and for whom completed data was available. Participants mean age was 70.4 years (SD = 6.2), included a majority of men (64.8%), with a mean of 15.4 years (SD = 2.9) of education and a mean diabetes duration of 16.3 years (SD = 9.3). 36.4% were insulin users and their mean HbA1c was 7.6 (SD = 1.4). The mean (SD) MoCA, standardized DSST and NeuroTrax GCS scores were 24.1 (3.4), 9.6 (3.0), and 98.3 (9.0), respectively (Table 1). Using the MoCA cut-off of 25 or above (20), 63 participants (51.6%) were cognitively intact. Utilizing a definition of intact cognitive function of above −1 SD (15) in the NeuroTrax GCS, 113 participants (92.6%) were deemed to be cognitively intact.

**Table 1:** Characteristics of Study Population (*N* = 122).

**Variable**	**Mean (SD)/Prevalence (%)/Median [IQR]**	**Variable**	**Mean (SD)/Prevalence (%)/Median [IQR]**
Age [years]	70.4 (6.2)	SDSCA diet	4.9 [3.6–6.2]
Gender [men]	79 (64.8)	SDSCA exercise	1.5 [0–2.5]
Education [years]	15.4 (2.9)	SDSCA blood-glucose testing	4.0 [1.0–7.0]
BMI [Kg/m^2^]	29.2 (4.7)	SDSCA medication	7.0 [7.0–7.0]
Diabetes duration [years]	16.3 (9.3)	SDSCA foot-care	3.4 [2.8–4.6]
Insulin users	44 (36.4)	PAQ score	5.5 (1.92)
HbA1c [% mmol/mol]	7.6 (1.4)	Carbohydrate/total [%]	46.3 (8.3)
Current Smokers	7 (5.7)	MoCA	24.1 (3.4)
HTN	85 (69.7)	DSST [norm]	9.6 (3.0)
IHD	43 (35.2)	VF phonetic fluency [z-score]	−0.4 (1.3)
CVD	19 (15.7)	VF semantic fluency [z-score]	−0.4 (1.33)
PVD	14 (11.6)	NeuroTrax GCS	98.28 (9.0)
Retinopathy	20 (16.9)	NeuroTrax memory	97.9 (14.6)
Nephropathy	29 (25.7)	NeuroTrax executive	98.9 (11.4)
Neuropathy	51 (42.1)	NeuroTrax attention	97.6 (11.0)
Dyslipidemia	114 (95.0)	NeuroTrax motor skills	98.8 (9.6)
Severe hypoglycemia [yes]	14 (11.5)		

*SD, Standard Deviation; IQR, Interquartile range; BMI, Body Mass Index; HbA1c, Hemoglobin A1c; HTN, Hypertension; IHD, Ischemic Heart Disease; CVD, Cerebrovascular Disease; PVD, Peripheral Vascular Disease; Severe Hypoglycemia, ever occurred hypoglycemic episode, requiring external assistance [yes/no]; SDSCA, Summary of Diabetes Self-Care Activities Assessment; PAQ, Physical Activity Questionnaire; Carbohydrate/total, carbohydrate/total energy consumption; MoCA, Montreal Cognitive Assessment; DSST, Digit Symbol Substitution Test; VF, Verbal Fluency Test; GCS, Global Cognitive Score*.

### Univariate Stepwise Regression Analysis

Table 2 presents the association between the different self-care capacity variables and the cognitive assessment tools: The MoCA and DSST were significantly associated with HbA1c; MoCA was also associated with the PAQ score and with severe hypoglycemia episodes. NeuroTrax GCS and domain specific scores were associated with the SDSCA exercise, blood-glucose testing, medication and foot-care domains.

**Table 2A:** Association between self-care capacity components and cognitive assessment tools.

	**Self-care capacity variables**									
	**SDSCA diet**		**SDSCA exercise**		**SDSCA blood-glucose testing**		**SDSCA medication**		**SDSCA foot-care**	
	***B***	***p***	***B***	***p***	***B***	***p***	***B***	***p***	***B***	***p***
MoCA			0.08	0.157						
NeuroTrax GCS			−0.13	**0.023**	0.29	**0.014**	0.06	**0.029**		
NeuroTrax memory			0.03	0.158	−0.09	**0.014**				
NeuroTrax executive			0.10	**0.001**	−0.14	**0.008**			−0.05	**0.006**
NeuroTrax motor Skills					−0.08	**0.049**				
NeuroTrax attention										
VF phonetic	−0.20	0.085	−0.29	0.059						
VF semantic			0.27	0.057	−0.49	**0.032**			−0.32	0.062
*R*^2^/adj. *R*^2^	0.025/0.016		0.150/0.098		0.166/0.231		0.88/0.152		0.122/0.162	

*SDSCA, Summary of Diabetes Self-Care Activities Assessment; MoCA, Montreal Cognitive Assessment; DSST, Digit Symbol Substitution Test; VF, Verbal Fluency Test; GCS, Global Cognitive Score*.

*p < 0.05 are bolded*.

**Table 2B:** Association between self-care capacity components and cognitive assessment tools.

	**Self-care capacity variables**							
	**HbA1c**		**PAQ**		**Carbohydrate/energy**		**Severe hypoglycemia**	
	***B***	***p***	***B***	***p***	***B***	***p***	***B***	***p***
MoCA	−0.01	**0.016**	0.15	**0.017**			−0.20	**0.009**
NeuroTrax GCS	0.01	0.109	−22.92	0.069	97.93	0.090		
NeuroTrax memory			5.73	0.069	−24.39	0.091		
NeuroTrax executive	−0.00	0.134	5.78	0.066	−24.32	0.092		
NeuroTrax motor Skills			5.75	0.068	−24.64	0.088		
NeuroTrax attention			5.70	0.071	−24.59	0.089		
DSST	−0.01	**0.033**						
*R*^2^/adj. *R*^2^	0.155/0.126		0.174/0.131		0.065/0.025		0.055/0.107	

*HbA1c, Hemoglobin A1c; PAQ, Physical Activity Questionnaire; Carbohydrate/total, carbohydrate/total energy consumption; Severe Hypoglycemia, ever occurred hypoglycemic episode, requiring external assistance [yes/no]; MoCA, Montreal Cognitive Assessment; DSST, Digit Symbol Substitution Test; VF, Verbal Fluency Test; GCS, Global Cognitive Score*.

*p < 0.05 are bolded*.

### Principal Component Analysis

**Figure 1:**
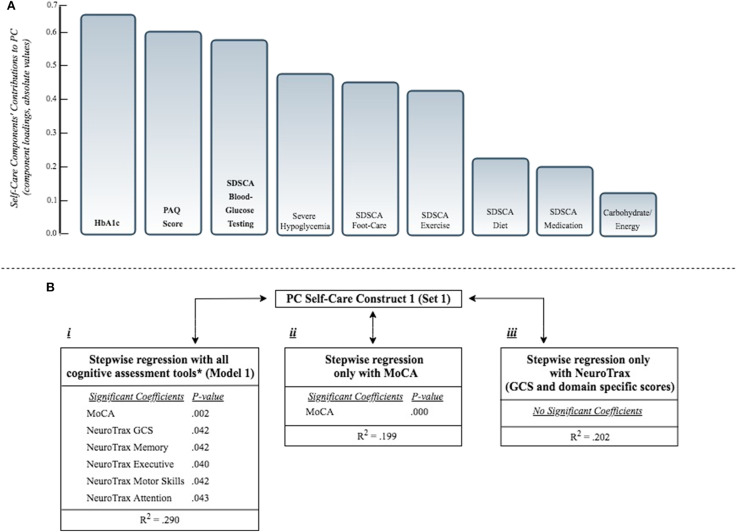
**(A)** Presents the self-care components' contributions to PC self-care construct 1 (from set1, including all self-care capacity variables; see Table 3). Components' contributions (Principal Component loadings, also known as eigenvectors) measure the correlation between the original self-care capacity variables and the Principal Component self-care construct. Component loadings ≥|0.5| are bolded (main contributors). **(B)** Presents the association of PC self-care construct 1 with different combinations of cognitive assessment tools, as independent variables, according to separate stepwise regression models: i. Stepwise regression model including all cognitive assessment tools (see Model 1, Table 4). ^*^All cognitive tools: NeuroTrax (GCS and domains specific scores), MoCA, DSST, VF. ii. Stepwise regression model including only the MoCA score. iii. Stepwise regression model including only the NeuroTrax (GCS and domain specific scores). All regression models were adjusted for age and gender. Only significant associations are presented (*P* < 0.05). PC, Principal Component; MoCA, Montreal Cognitive Assessment; GCS, Global Cognitive Score; DSST, Digit Symbol Substitution Test; VF, Verbal Fluency Test; HbA1c, Hemoglobin A1c; SDSCA, Summary of Diabetes Self-Care Activities Assessment (possible range 0-7); PAQ, Physical Activity Questionnaire; Carbohydrate/Energy, carbohydrate/total energy consumption; Severe hypoglycemia, ever occurred hypoglycemic episode, requiring external assistance [yes/no].

Two sets of self-care capacity variables were used to the create the Principal Component self-care constructs (PCs), as summarized in Table 3:

- Set 1 included all self-care capacity variables. The PCA using this set of variables revealed two PCs (PC1 & PC2) with eigenvalues >one, accounting together for 39% of the total variance. In this analysis, the first PC (PC1) was mainly composed (PCs loadings ≥ |0.5|) of HbA1c, SDSCA blood-glucose testing and the PAQ score (Figure 1A). The second PC (PC2) was mainly composed of SDSCA diet, exercise and the PAQ score.
- Set 2 included all self-care capacity variables that were not strongly associated with the PCs constructed using set 1 (PCs loadings < |0.6|): Carbohydrate consumption/total caloric intake, severe hypoglycemia, SDSCA blood-glucose testing, medication and foot-care. The analysis using this set of variables (set 2) constructed two PCs, explaining together 49% of the total variance. PC1 was mainly composed of SDSCA blood-glucose testing and foot-care, while PC2 was mainly composed of SDSCA medication.

**Table 3:** Principal components loadings and variance explained.

**Self-care capacity variables**	**Component loadings**			
	**Set 1**		**Set 2**	
	**PC1**	**PC2**	**PC1**	**PC2**
HbA1c	**0.64**	−0.11	−	−
SDSCA diet	0.23	**−0.66**	−	−
SDSCA exercise	−0.44	**−0.69**	−	−
SDSCA blood-glucose testing	**0.59**	−0.34	**−0.76**	−0.26
SDSCA medication	0.20	−0.49	−0.37	**0.67**
SDSCA foot-care	0.46	−0.22	**−0.66**	0.35
PAQ score	**−0.60**	**−0.53**	−	−
Carbohydrate/energy	0.12	0.22	0.15	0.48
Severe hypoglycemia	0.49	−0.05	−0.42	−0.48
Proportion of variance explained (%)	20.92	18.34	26.97	22.0
Cumulative proportion of variance explained (%)	39.26		48.97	

*Component loadings (Principal Component loadings, also known as eigenvectors) measure the correlation between the original self-care capacity variables and the Principal Components self-care constructs. Component loadings ≥|0.5| are bolded (main contributors)*.

*HbA1c, Hemoglobin A1c; SDSCA, Summary of Diabetes Self-Care Activities Assessment; PAQ, Physical Activity Questionnaire; Carbohydrate/total, carbohydrate/total energy consumption; Severe Hypoglycemia, ever occurred hypoglycemic episode, requiring external assistance [yes/no]*.

### Principal Components Stepwise Linear Regression Analysis

Using the stepwise procedure, several regression models were conducted, examining the association between each of the 4 PCs and all cognitive assessment tools. Model 1 explained a relatively high percentage of the variability (*R*^2^ = 0.29) of PC self-care construct 1. Among the cognitive scores included in this model, the MoCA and the NeuroTrax (GCS and domain specific) scores were significantly associated with this construct (Figure 1B). Model 2, 3 and 4 explained a relatively low percentage of the variability of the respective PC self-care constructs (*R*^2^ = 0.036, 0.177, 0.037; Model 2, Model 3, Model 4, respectively). In model 3, DSST and VF Phonetic scores were significantly associated with PC1 (set 2). In model 4, NeuroTrax GCS was the only significant score to be associated with PC2 (set 2) (Table 4).

**Table 4:** The association between principal components self-care constructs (PCs) and cognitive assessment tools.

	**Set 1**				**Set 2**			
	**Model 1 (PC1)**		**Model 2 (PC2)**		**Model 3 (PC1)**		**Model 4 (PC2)**	
	***B***	***p***	***B***	***p***	***B***	***p***	***B***	***p***
MoCA	−0.14	**0.002**						
NeuroTrax GCS	17.12	**0.042**					0.02	**0.034**
NeuroTrax memory	−4.27	**0.042**						
NeuroTrax executive	−4.31	**0.040**	−0.02	0.068				
NeuroTrax motor Skills	−4.29	**0.042**						
NeuroTrax attention	−4.26	**0.043**						
DSST					0.08	**0.035**		
VF Phonetic			0.16	0.105	0.21	**0.017**		
VF Semantic	−0.18	0.068						
*R*^2^/adj. *R*^2^	0.290/0.246		0.036/0.020		0.177/0.149		0.037/0.029	

*Set 1 includes all self-care capacity variables. Set 2 includes carbohydrate consumption/total caloric intake, severe hypoglycemia, SDSCA blood-glucose testing, medication and foot-care. P < 0.05 are bolded*.

*PC, Principal Component; MoCA, Montreal Cognitive Assessment; GCS, Global Cognitive Score; DSST, Digit Symbol Substitution Test; VF, Verbal Fluency Test; HbA1c, Hemoglobin A1c; SDSCA, Summary of Diabetes Self-Care Activities Assessment (possible range 0-7); PAQ, Physical Activity Questionnaire; Carbohydrate/Energy, carbohydrate/total energy consumption; Severe hypoglycemia, ever occurred hypoglycemic episode, requiring external assistance [yes/no]*.

In a model including only the MoCA score, after adjusting for age and gender, a statistically significant association was found between PC self-care construct 1 and the MoCA score (*p* < 0.000, *R*^2^ = 0.19). The same was not found in a model that included the NeuroTrax GCS and specific domain scores (Figure 1B).

## Discussion

In this analysis of 122 older people with diabetes, several cognitive tools were found to be associated with self-care capacity indices. When unifying these variables into a self-care capacity construct, a significant association was found between this construct and the MoCA and NeuroTrax scores. In a model including only the MoCA score, a statistically significant association was found between this self-care construct and the MoCA score. The same was not found in a model including only the NeuroTrax GCS and specific domain scores (Figure 1B).

Several professional organizations have recommended cognitive screening for older people with diabetes (8–12). This is in light of the fact that cognitive ability affects self-care capacity. Several cognitive assessment tools are suggested by these guidelines, but they do not specify which one should be used. This study demonstrates the association between the MoCA score and self-care capacity, suggesting that the MoCA may be useful as an adjunct to assessment of self-care capacity in this population, in whom cognitive impairment may interfere with diabetes self-management.

The MoCA is a one-page 30-points test, administered in 10 min. Multiple cognitive domains are assessed by this tool, including memory, language, orientation, executive function, praxis, visuospatial abilities, and attention. This brief cognitive screening instrument that has a short administration time and is freely available, had been translated into numerous languages. The MoCA demonstrates an excellent test-retest reliability. It has been validated as a screening tool for cognitive impairment, demonstrating positive and negative predictive values for MCI and Alzheimer's Disease, with a suggested cut-off score above 26 designating normal cognitive function (20). Moreover, this instrument is among the few brief cognitive screening instruments that have been validated in a population-based cohort (32).

The MoCA has demonstrated an ability to detect cognitive impairment that may affect self-care in other disease states. Among patients with chronic heart failure, the MoCA has shown to be more sensitive than other screening tools, in identifying cognitive impairment that has potential to impact the ability to make self-care decisions (33). The MoCA was found to be more sensitive than the frequently used Mini-Mental State Examination (MMSE) when screening for MCI in other chronic diseases, such as Parkinson's disease (34), as well as in other medical conditions, for example in post-stroke patients (35).

In older people with diabetes, the MoCA may be a useful tool, as it assesses different cognitive domains which are important for planning, executing and foreseeing consequences of decisions (36), as well as for self-care decisions. Patients with diabetes tend to perform worse than patients without diabetes, when measuring cognitive function using brief cognitive tests. A systematic review, examining the utility of brief cognitive tests in this population, found the MoCA to be superior to other commonly used tests for detecting MCI in elderly patients with type 2 diabetes (37).

This study has several limitations. First, the use of a convenience sample of individuals who were referred by health care professional or self-referred because of difficulties in managing their disease. The awareness to those difficulties may suggest relatively high self-care abilities and therefore may limit the ability to generalize these results. Second, the cross-sectional design of this study does not allow assessment of temporality of the relationship. Third, some of the instruments used (such as the PAQ) has not been validated for the older population. Fourth, the study aimed to elucidate cognitive tools that are associated with self-care. Self-care is a construct that does not have a measurable definition, thus limiting our ability to define the relationship between it and the cognitive assessment tools. In order to overcome this, a multitude of variables that compose the self-care construct were considered and analyzed together, using the PCA method. This method may be valuable to explore the multidimensional construct of self-care capacity.

Considering the projected prevalence of diabetes as the population is aging, tailoring treatment require efficient cognitive evaluation tools. The MoCA, previously validated as a brief cognitive impairment screening tool, may be useful as an adjunct to assess the self-care capacity of older individuals with diabetes. Future studies are needed to assess its use in different types of diabetes (like type 1), to understand the role of mediating factors (like cognitive reserve) and if using the MoCA in the clinic, as a screening tool, may improve tailoring of treatment plan.

## Data Availability Statement

The datasets generated for this study are available on request to the corresponding author.

## Ethics Statement

The studies involving human participants were reviewed and approved by Ethical Committee of the Sheba Medical Center. The patients/participants provided their written informed consent to participate in this study.

## Author Contributions

NG, MA, OG-T, TY, and RN contributed to acquisition and interpretation of data. TC-Y and NM contributed to acquisition, interpretation, analysis of data, and drafting this manuscript. TC-Y also made substantial contribution to conception and design of the research described. All authors have read and approved the final manuscript.

## Conflict of Interest

The authors declare that the research was conducted in the absence of any commercial or financial relationships that could be construed as a potential conflict of interest. The handling Editor declared a past co-authorship with the author TC-Y.
